# The reliability of an adolescent dietary pattern identified using reduced-rank regression: comparison of a FFQ and 3 d food record

**DOI:** 10.1017/S0007114514001111

**Published:** 2014-05-22

**Authors:** Geeta Appannah, Gerda Karolien Pot, Therese Anne O'Sullivan, Wendy Hazel Oddy, Susan Ann Jebb, Gina Leslie Ambrosini

**Affiliations:** 1 MRC Human Nutrition Research, Cambridge, UK; 2 Department of Nutrition and Dietetics, Faculty of Medicine and Health Sciences, Universiti Putra Malaysia, Serdang, Malaysia; 3 Diabetes and Nutritional Sciences Division, School of Medicine, King's College London, London, UK; 4 School of Exercise and Health Science, Edith Cowan University, Joondalup, WA, Australia; 5 Telethon Institute for Child Health Research, University of Western Australia, Perth, WA, Australia; 6 School of Population Health, University of Western Australia, Perth, WA, Australia

**Keywords:** Dietary patterns, Reliability, Adolescents, Raine Study

## Abstract

Despite the increasing use of dietary patterns (DP) to study diet and health outcomes, relatively few studies have examined the reliability of DP using different dietary assessment methods. Reduced-rank regression (RRR) is an emerging statistical method that incorporates *a priori* information to characterise DP related to specific outcomes of interest. The aim of the present study was to compare DP identified using the RRR method in a FFQ with those in a 3 d food record (FR). Participants were 783 adolescents from the Western Australian Pregnancy (Raine) Cohort Study who completed both a FFQ and FR at 14 years of age. A similar ‘energy-dense, high-fat and low-fibre’ DP was identified in the FFQ and FR that was characterised by high intakes of processed meat and sugar-sweetened beverages, and low intakes of vegetables and fresh fruit. Nutrient profiles for this DP were consistent in the FFQ and FR. Pearson's correlation coefficient between participants' *z*-scores for the DP identified in the FFQ and FR was 0·35 for girls and 0·49 for boys (*P*< 0·05). The mean difference between DP *z*-scores derived from the FFQ and FR was − 0·08 (95 % CI − 0·21, 0·04) for girls and − 0·05 (95 % CI − 0·17, 0·07) for boys. The 95 % limits of agreement were − 2·55 to 2·39 for girls and − 2·52 to 2·41 for boys. These findings suggest that very similar DP may be identified and their *z*-scores show modest agreement when applying the RRR method to dietary intake data collected from adolescents using a FFQ or FR.

Dietary pattern analysis is a useful method for studying the role of diet in relation to health outcomes or disease risk. Dietary patterns have some advantages over the analysis of single nutrients or foods as they consider the total diet and the cumulative and interactive effects of foods and nutrients eaten together, hence representing a more holistic perspective^(^ ^1^ ^)^. Empirical dietary patterns are identified using statistical dimension-reduction techniques, which can identify a small number of underlying constructs, or dietary patterns, from a large number of dietary variables. One such method is reduced-rank regression (RRR), which combines *a priori* information with exploratory statistics to identify dietary patterns related to specific outcomes of interest. This method has been applied in studies examining dietary patterns in relation to various outcomes, including diabetes, obesity and heart disease^(^ ^2^ ^–^ ^4^ ^)^

Most studies investigating dietary patterns have employed a FFQ to estimate dietary intakes, owing to their lower cost and ease of administration compared with a more detailed food diary method. However, FFQ are often criticised for their lack of precision, particularly when applied in child or adolescent populations^(^ ^5^ ^)^. Therefore, it is very important to assess whether the limitations of a FFQ affect their ability to describe dietary patterns. To date, no studies have compared RRR-derived dietary patterns using different dietary assessment methods in children or adolescents. Therefore, the present study set out to assess (1) whether the same dietary pattern, hypothesised to be associated with the risk of obesity in children and adolescents, could be identified in both a FFQ and 3 d food record (FR), and (2) whether dietary pattern *z*-scores from each dietary assessment method are in agreement.

## Materials and methods

### Study population

Participants in the present study were adolescent offspring in the Western Australian Pregnancy Cohort (Raine) Study^(^ ^6^ ^)^. In brief, 2900 pregnant women were recruited into a trial at King Edward Memorial Hospital (Perth, Western Australia) to examine ultrasound imaging from 1989 to 1991. A total of 2868 children born to 2804 mothers remained with the study and subsequently formed the Raine cohort. These children were followed up at regular intervals, i.e. 1, 2, 3, 5, 8, 10, 14 and 17 years of age. The present analysis used data collected at the 14-year follow-up, when both a FFQ and FR were administered. Of the 2868 baseline sample, 2337 (82 %) adolescents were eligible for a follow-up at 14 years of age, while 152 (5 %) were lost to follow-up, 348 (12 %) had withdrawn from the study and thirty-one (1 %) were deceased. Ethical approval for the study was obtained from the ethics committees of King Edward Memorial Hospital and Princess Margaret Hospital for Children. Adolescents and their parent or guardian gave informed written consent.

### Dietary assessments

A 227-item semi-quantitative FFQ developed by the Commonwealth Scientific and Industrial Research Organisation (CSIRO) was used to estimate habitual dietary intakes over the previous year. Parents of adolescents completed the FFQ with their child at the 14-year follow-up^(^ ^7^ ^)^. For each food item, the average frequency of consumption over the past year was recorded as ‘never’, ‘rarely’, ‘times a month’, ‘times a week’ or ‘times a day’. The selected frequency category for each food item was then converted to a daily intake (g) and linked by the CSIRO with the Australian food composition database to estimate daily nutrient intakes^(^ ^8^ ^)^. This FFQ was evaluated; relative to a 3 d FR, the FFQ correctly ranked most individuals according to their nutrient intakes at 14 years of age^(^ ^9^ ^)^. All food and beverage intakes (*n* 227 items) in the FFQ were assigned to forty-six predefined food groups based on nutrient profiles or culinary usage, and their hypothesised contribution to diet–disease relationships^(^ ^10^ ^)^. A total of 1631 adolescents completed the FFQ at the 14-year follow-up. Of these, 1611 reported plausible energy intakes (>3000 and < 20 000 kJ/d) and were included in the analysis.

Participants were also requested to complete a 3 d FR with parental assistance at the 14-year follow-up. Participants were not required to complete their FR on a certain number of week days or weekend days. Of the 1286 adolescents who agreed, 962 returned a completed FR. Where any of the 3 d recorded was noted by the respondent as not typical or unrepresentative of their usual diet, the FR was excluded from the analysis. A total of 822 FR were classified as representative of usual eating habits and included in the analysis. All FR were coded by a dietitian for nutrient analysis using the Australian food composition database^(^ ^8^ ^)^. A total of 4400 foods were recorded in the 822 FR, and each food was allocated to one of the forty-six predefined food groups described above. Mixed dishes were disaggregated into main constituents before food group coding.

Using the ‘Goldberg cut-off’ method, dietary misreporting was calculated based on the ratio of energy intake:energy expenditure (EI:EE)^(^ ^11^ ^)^. A 95 % CI was calculated for EI:EE to take account of the potential variation in the estimates of EI and EE. Participants were categorised as under-reporters if their EI:EE was < 95 % CI, over-reporters if their EI:EE was >95 % CI and plausible reporters if their EI:EE was within the 95 % CI. Dietary misreporting was calculated only using the dietary intake data estimated from the FFQ.

### Dietary pattern analysis

The partial least-squares procedure with a RRR option in SAS (SAS Institute, Inc.) was applied to derive the dietary patterns. In brief, the RRR method identifies linear combinations of weighted food intakes, or patterns in food intakes, that explain the maximum variation in a set of response variables, which are hypothesised to be on the pathway between food intake and a health outcome of interest^(^ ^12^ ^)^. In the present study, the RRR model included intakes of the forty-six predefined food groups (g/d) as predictor variables and intakes of three dietary variables (dietary energy density (DED), percentage of energy from total fat intake and fibre density) as response variables. These response variables were linked with obesity and were of interest in relation to the risk of obesity in the Raine Study^(^ ^3^ ^,^ ^9^ ^,^ ^13^ ^)^. In 2003, the WHO classified foods with high DED and low fibre density to be important predictors of obesity in adults^(^ ^13^ ^)^. Furthermore, a dietary pattern characterised by high DED and percentage of energy from total fat intake and low intakes of fibre in childhood and adolescence has been shown to be prospectively associated with greater adiposity in a UK pregnancy cohort^(^ ^3^ ^)^. DED was calculated by dividing total food energy (kJ) by total food weight (g) excluding beverages because they may disproportionately influence total DED values^(^ ^14^ ^,^ ^15^ ^)^. Fibre density was expressed as absolute intake of fibre (g/d) divided by total daily energy intake (MJ). Percentage of energy from total fat intake was calculated by dividing total energy intake from fat (kJ) by total energy intake (kJ) and then multiplying by 100.

Separate RRR analyses were applied to the FFQ and FR data. Each study participant received a *z*-score for the dietary pattern identified in the FFQ and FR, discriminating how strongly their dietary intakes corresponded with the dietary patterns. These *z*-scores were then categorised into quartiles to enable comparisons between the two dietary assessment methods. Separate RRR analyses were conducted for boys and girls, although their dietary patterns were similar, and therefore dietary patterns for the whole sample are reported here.

### Statistical methods

Dietary patterns identified in the FFQ and FR were compared using three methods. First, the product of the dietary patterns, their nutrient profiles, was compared. Pearson's correlation coefficient was used to compare nutrient intakes estimated from the FR (as reference) with dietary pattern *z*-scores derived from the FFQ and FR. The nutrient intakes estimated from the FR were adjusted for total energy intake (kJ) using the residual method^(^ ^16^ ^)^. Key nutrients of interest included carbohydrate, protein, total fat, fibre, sugars, saturated fat, monounsaturated fat, polyunsaturated fat, cholesterol, Na, Ca, K, Mg, folate, Fe, thiamin, niacin, riboflavin, Zn, retinol, vitamin A and vitamin C.

Second, study participants' *z*-scores for the dietary pattern identified in the FFQ and FR were compared using the partial Pearson's correlation coefficient, adjusted for dietary misreporting. Although correlations have been widely used in many studies, they only measure the strength of a relationship between two variables and not the agreement between them^(^ ^17^ ^)^. To examine the exact agreement between dietary pattern *z*-scores derived from the FFQ and FR, Bland–Altman plots were used^(^ ^17^ ^)^. The plots showed the difference between each individual's *z*-scores derived from FFQ and FR against their averages^(^ ^17^ ^)^. The mean difference and 95 % limits of agreement (LOA, calculated as mean differences and ± 2 sd) were used to summarise agreement at the population level. For a normally distributed variable, the LOA describes the range containing 95 % of all individual's differences between their FFQ and FR *z*-scores; the wider the LOA, the weaker the agreement^(^ ^17^ ^)^. The Bland–Altman plots also illustrate whether agreement between the FFQ and FR varies with the magnitude of *z*-scores by showing the fitted regression line (slope) between differences and averages.

## Results

### Characteristics of the study population

A total of 783 adolescents completed both the FFQ and 3 d FR at 14 years of age. Participation was similar for girls (49 %) and boys (51 %). Of 783 adolescents, the proportion of dietary under-reporters, plausible reporters and over-reporters was 36, 57 and 7 %, respectively. Adolescents who were overweight or obese were more likely to be dietary under-reporters compared with other misreporting categories (57 *v*. 43 %). Other characteristics of cohort members who completed both a FFQ and FR at the 14-year follow-up were previously compared^(^ ^9^ ^)^. In summary, those included in the present analysis had higher levels of maternal education, and were slightly less likely to be overweight or from low-income families^(^ ^9^ ^)^.

### Dietary patterns identified in the FFQ and 3 d food record

As three response variables were included in the RRR analyses, three dietary patterns were identified. The first dietary pattern identified in the FFQ and FR was positively correlated with DED and percentage of energy from total fat intake, but negatively correlated with fibre density. This dietary pattern explained the most variation in all response variables in the FFQ and FR (53 and 46 %, respectively). The subsequent two dietary patterns explained little additional variation; 15 and 12 % for the second dietary pattern and only 7 and 8 % for the third dietary pattern, in the FFQ and FR, respectively. These two dietary patterns were not as easily interpretable and were not hypothesised to be associated with the risk of obesity. Therefore, only the first dietary pattern was taken forward for further analysis.

**Fig. 1:**
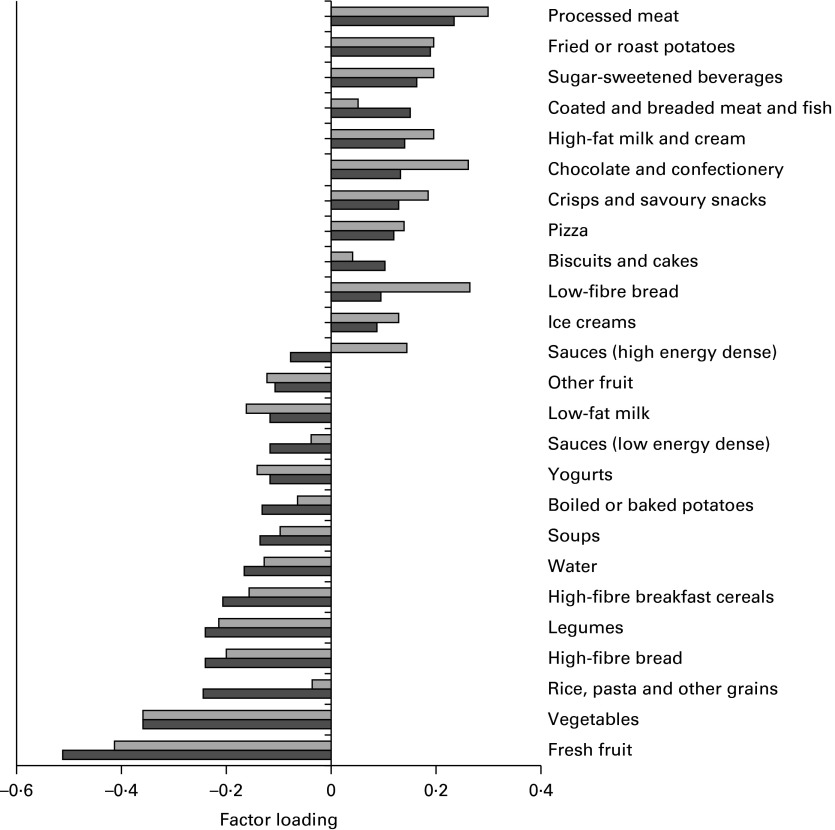
Factor loadings for an ‘energy-dense, high-fat and low-fibre’ dietary pattern identified using the FFQ (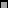) and 3 d food record (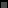) (Raine Study). Food groups with very small factor loadings ( < |0·10|) were excluded from the graph for brevity. These included butter and animal fats, margarine and vegetable oils, eggs and egg dishes, other bread products, other breakfast cereals, cereal-based mixed meals, puddings, spreads, meat and poultry, mixed meat dishes, fish, meat substitutes, fried vegetables, mixed vegetable dishes, nuts and seeds, low-energy beverages, fruit juice, hot and powdered drinks.

Fig. 1 shows the factor loadings for the ‘energy-dense, high-fat and low-fibre’ dietary pattern identified using the FFQ and FR. Intakes of foods with a positive factor loading increased the dietary pattern *z*-score, while intakes of foods with a negative factor loading decreased the *z*-score. The ‘energy-dense, high-fat and low-fibre’ dietary pattern identified in the FFQ was strongly characterised by high intakes of processed meat, chocolate and confectionery, low-fibre bread, sugar-sweetened beverages, full-fat milk, and crisps and savoury snacks (Fig. 1). Similarly, the ‘energy-dense, high-fat, low-fibre’ dietary pattern identified in the FR was also strongly characterised by high intakes of processed meat, sugar-sweetened beverages, full-fat milk, and crisps and savoury snacks, in addition to fried/roasted potatoes (chips) and coated/breaded meat and fish. In both the FFQ and FR, the dietary pattern was strongly negatively associated with intakes of vegetables, fresh fruit, high-fibre bread, legumes and high-fibre breakfast cereals. Notably, factor loadings for fresh fruit and vegetables were the strongest of all the food groups (almost double the loading for processed meat) in both the FFQ and FR (Fig. 1).

Factor loadings in the FR were generally lower than those in the FFQ. Some notable differences in factor loadings were observed for rice, pasta and other grains, high-energy-dense sauces and low-fibre bread (Fig. 1). However, despite the different factor loadings observed for some food groups, the majority were similar in their ranking and associations (Fig. 1).

### Nutrient profiles

**Fig. 2:**
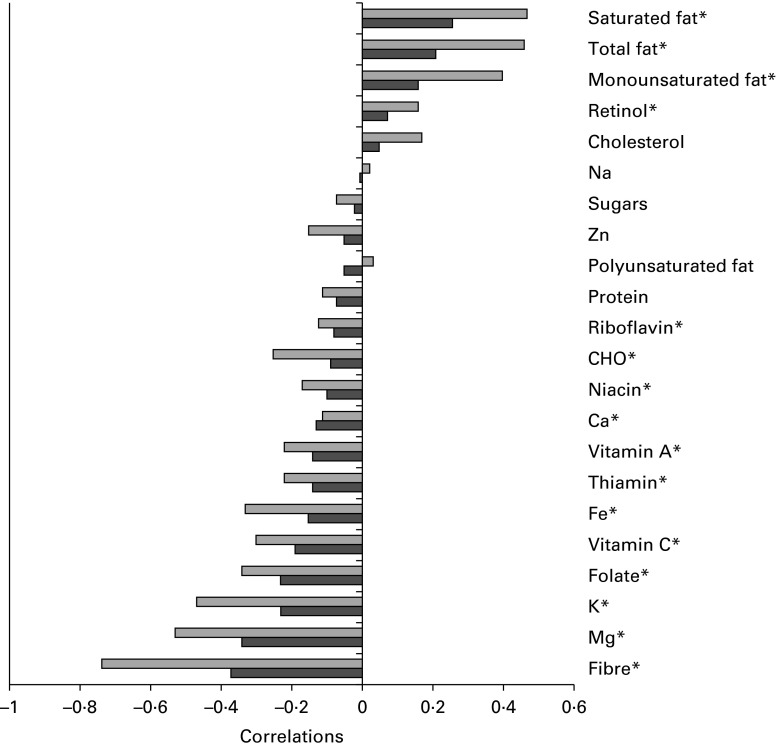
Correlations between nutrient intakes estimated from the 3 d food record (FR, 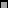) and dietary pattern *z*-scores derived from the FFQ (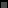) and 3 d FR (Raine Study). Nutrient intakes were estimated from 3 d FR and adjusted for total energy intake using the residual method^(^ ^14^ ^)^. * *P*< 0·05 for the FFQ. CHO, carbohydrate.

The participants' *z*-scores for the ‘energy-dense, high-fat and low-fibre’ dietary pattern identified in both the FFQ and FR were positively correlated with intakes of saturated fat, total fat, monounsaturated fat, retinol, cholesterol and negatively correlated with carbohydrate, protein, fibre, Mg, K, folate, vitamin C, Fe, thiamin, vitamin A, Ca, niacin, riboflavin and Zn, as estimated from the FR (Fig. 2). As expected, correlations were weaker between the FFQ and FR; however, the directions of associations were consistent.

### Correlations and agreement between dietary pattern z-scores

**Fig. 3:**
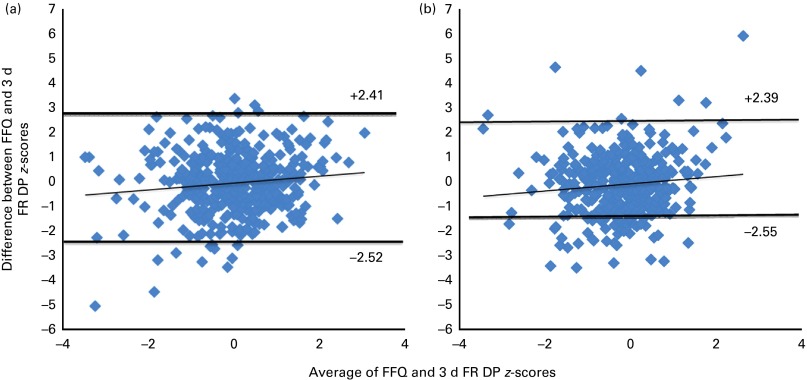
Bland–Altman plots of dietary pattern (DP) *z*-scores derived from the FFQ and 3 d food record (FR) for (a) boys and (b) girls (Raine Study). 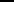 Represents 95 % limits of agreement between DP *z*-scores derived from the FFQ and 3 d FR. A regression line (slope) was fitted by the regression of differences in DP *z*-scores against their averages: (a) *r* 0·49, *P*< 0·01; (b) *r* 0·35, *P*< 0·01. (A colour version of this figure can be found online at http://www.journals.cambridge.org/bjn).

Pearson's correlation coefficient between the dietary pattern *z*-scores derived from the FFQ and FR was 0·35 for girls and 0·49 for boys (*P*< 0·05) after adjusting for dietary misreporting. Moderate agreement was observed between the dietary pattern *z*-scores derived from the FFQ and FR with a non-significant mean difference of − 0·08 (95 % CI − 0·21, 0·04) for girls and − 0·05 (95 % CI − 0·17, 0·07) for boys (Fig. 3). The 95 % LOA were similar for girls ( − 2·55, 2·39) and boys ( − 2·52, 2·41) (Fig. 3). However, the significant slope between the averages and differences in dietary pattern *z*-scores (*r* 0·49 for boys, *r* 0·35 for girls) indicated that agreement between the FFQ and 3 d FR decreased as the dietary pattern *z*-scores increased.

## Discussion

In the present study of a large sample of adolescents, a similar ‘energy-dense, high-fat and low-fibre’ dietary pattern was identified using the RRR method in a FFQ and FR, and there was modest agreement between the dietary pattern *z*-scores.

No published studies have previously reported comparisons between RRR-derived dietary patterns in a FFQ and FR in adolescents. However, exploratory principal components analysis was previously applied in the Raine Study to identify ‘Healthy’ and ‘Western’ dietary patterns in the FFQ, which were compared with those in the FR at 14 years of age^(^ ^18^ ^)^. The correlations for the ‘Healthy’ and ‘Western’ dietary patterns were observed to be 0·47 and 0·34 for boys and 0·42 and 0·38 for girls, respectively^(^ ^18^ ^)^. These correlation coefficients compare well with those in the present study despite the fact that different statistical methods were used to identify the dietary patterns. In adults, correlations between principal components analysis-derived dietary pattern *z*-scores identified using a FFQ and 1-week diet records ranged from 0·34 to 0·73^(^ ^19^ ^,^ ^20^ ^)^. Similarly, dietary patterns identified using principal components analysis among pregnant women in the UK also suggested correlation coefficients to be ranging between 0·35 and 0·67 for the dietary patterns derived from a FFQ and 4 d food diaries^(^ ^21^ ^)^. Furthermore, differences in factor loadings for a few food groups have also been reported by some of these studies^(^ ^5^ ^,^ ^18^ ^,^ ^19^ ^)^.

Although the key foods and their factor loadings for the ‘energy-dense, high-fat and low-fibre’ dietary pattern were similar for the FFQ and FR, some differences were noted, in particular, for high energy-dense sauces, low-fibre bread, rice, pasta and other grains. Some variations might be expected given the differences in dietary assessment methods used in the present study. The FFQ was designed to capture habitual dietary intakes for the past 12 months, whereas the FR only captured foods eaten (and recorded) over a 3 d period. Therefore, a FR may not capture food items consumed infrequently. Furthermore, variations in the disaggregation of mixed dishes in the FR may have contributed to the differences in factor loadings for the ‘rice and pasta’ food group. With the use of principal components analysis-derived dietary patterns, an earlier study of this cohort has also reported that factor loadings for all food groups in the FR were generally weaker than those observed in the FFQ at 14 years of age^(^ ^18^ ^)^. Similar findings were also observed among American men in a study by Hu *et al.* ^(^ ^19^ ^)^. While the RRR method incorporates *a priori* information in the form of response variables, it is also partly an exploratory statistical method that depends on the sample correlation matrix. Hence, some variations would be expected in factor loadings regardless of the differences in dietary assessment methods.

The correlations between the dietary patterns derived from the FFQ and FR were observed to be higher in boys than in girls. These results were not unforeseen as studies among adolescents have shown greater inconsistency in the recording of food intakes among adolescent girls than among boys^(^ ^5^ ^,^ ^18^ ^)^. However, there may be differences between the two variables across their range of values even if these variables are highly correlated. In contrast, mean agreement and 95 % LOA are better indicators of exact agreement between two different dietary assessment methods^(^ ^17^ ^,^ ^18^ ^)^. In the present study, the 95 % LOA between the dietary pattern *z*-scores derived from the FFQ and FR were comparable with the earlier analysis in the Raine Study in which minor differences were observed using the 95 % LOA between boys and girls in ‘Healthy’ and ‘Western’ dietary patterns identified from the FFQ and FR^(^ ^18^ ^)^.

There are some limitations in the present study. First, the analysis was confined to those adolescents who completed both the FFQ and FR (*n* 783). The observed differences between these participants and the remainder of the cohort may limit the generalisability of these findings. Nonetheless, the same ‘energy-dense, high-fat and low-fibre’ dietary pattern was identified in the whole cohort (*n* 1611) using the FFQ at 14 years of age. Furthermore, while FFQ are often criticised for their measurement error, it is important to note that a FR is not error-free. For example, they may be prone to dietary under-reporting and may not reflect habitual dietary intake, as foods eaten infrequently may not be captured^(^ ^5^ ^)^. We have, however, taken steps to minimise the impact of dietary under-reporting and unrepresentative diary recording days.

The strengths of the present study include a sample size which is larger than that included in most reliability studies and good response rates, with 70 and 75 % of adolescents who were eligible for follow-up, completing a FFQ and FR, respectively, at 14 years of age. Adolescents tend to show a greater variation in their dietary intakes than younger children and adults; therefore, assessing their usual food intakes is challenging^(^ ^5^ ^)^. However, we attempted to control for dietary misreporting, and this was considered as a potential confounder in the present analysis. Additionally, the FFQ has been evaluated in this cohort and been shown to correctly rank a reasonable proportion of nutrient intakes relative to this FR at 14 years of age^(^ ^9^ ^)^.

### Conclusions

In this adolescent cohort, a comparable ‘energy-dense, high-fat and low-fibre’ dietary pattern was identified using the RRR method in a FFQ and FR. The present study supports the use of a FFQ to study the relationships between a RRR-derived dietary pattern and health outcomes in Australian adolescents.
